# The Role of IL-17-Producing Cells in Cutaneous Fungal Infections

**DOI:** 10.3390/ijms22115794

**Published:** 2021-05-28

**Authors:** Yu Sawada, Ayako Setoyama, Yumiko Sakuragi, Natsuko Saito-Sasaki, Haruna Yoshioka, Motonobu Nakamura

**Affiliations:** Department of Dermatology, University of Occupational and Environmental Health, Iseigaoka, Yahatanishi-ku, Kitakyushu, Fukuoka 807-8555, Japan; ayako-s@med.uoeh-u.ac.jp (A.S.); y-sakuragi@med.uoeh-u.ac.jp (Y.S.); natsuko-saito@med.uoeh-u.ac.jp (N.S.-S.); haruna-yoshioka@med.uoeh-u.ac.jp (H.Y.); motonaka@med.uoeh-u.ac.jp (M.N.)

**Keywords:** IL-17, Th17, antimicrobial peptide, fungal infection, skin

## Abstract

The skin is the outermost layer of the body and is exposed to many environmental stimuli, which cause various inflammatory immune responses in the skin. Among them, fungi are common microorganisms that colonize the skin and cause cutaneous fungal diseases such as candidiasis and dermatophytosis. The skin exerts inflammatory responses to eliminate these fungi through the cooperation of skin-component immune cells. IL-17 producing cells are representative immune cells that play a vital role in anti-fungal action in the skin by producing antimicrobial peptides and facilitating neutrophil infiltration. However, the actual impact of IL-17-producing cells in cutaneous fungal infections remains unclear. In this review, we focused on the role of IL-17-producing cells in a series of cutaneous fungal infections, the characteristics of skin infectious fungi, and the recognition of cell components that drive cutaneous immune cells.

## 1. Introduction

The abundance of environmental stimuli is constitutively surrounding human body. The skin is an organ located in the outermost layer of the human body. Because of this characteristics, the skin is easily exposed to various external environmental stimuli [1,2], such as ultraviolet light, chemicals, and allergens in daily life [3,4,5]. Among these environmental factors, microorganisms are representative external environmental agents that exert both harmful and beneficial effects on human skin health. *Streptococcus aureus* and *Propionibacterium acnes* are major colonizing microorganisms in the epidermis and exhibit virulence to exacerbate cutaneous inflammatory disease, whereas some microorganisms have a beneficial impact on the skin [6]. Therefore, the knowledge of these microorganisms on the skin is important for clinicians.

Fungi are a group of eukaryotic organisms including yeasts, and are capable of infecting human skin with immunocompromised hosts, resulting in the development of fungal skin diseases [7]. Fungal infections are localized on the skin surface and hair follicles; they are sometimes insensitive to current treatment because of their characteristics of invasive and persistent infection facilitated by the immunosuppressive action of their cell components [8].

The skin exerts immune reactions to defend against external environmental stimuli [9]. Although fungi colonize the epithelial surface, keratinocytes, the main epidermal component cells, drive inflammatory reactions after the recognition of specific pattern of the pathogens. The skin counters harmful microorganisms through the interaction of various cells in the skin by neutrophil infiltration, epidermal proliferation, and antimicrobial peptide production for host protection [10]. Further, dermatophytes initiate an adaptive immune response that protects against re-infection [11]. To exert antimicrobial action against microorganisms in the skin, various cells cooperate to promote bactericidal action using different approaches to eliminate skin-colonized microorganisms. Among these cells, Th17 cells secrete IL-17 and IL-22 for enhancing antimicrobial peptide production and immune cell function against microorganisms. IL-17 cytokines play a vital role in the cutaneous host defense against microorganisms such as fungi [12]. In human dermatophytosis, exacerbation of skin infection has been observed in patients with a deficiency of the IL-17 pathway [13]. Because of the current development of cytokine-targeted treatment, IL-17-targeted treatment has also demonstrated the importance of IL-17 in the skin. For instance, treatment with anti-IL-17 antibody results in a high frequency of superficial fungal infection in the skin, which sometimes becomes intractable with topical antifungal treatment.

A limited number of reports have explored the contribution of IL-17 to fungal skin infection. Thus, the detailed mechanisms underlying the beneficial effects of IL-17 on a series of skin fungal infections remain unclear. In this review, we focused on the involvement of IL-17-producing cells in the skin in the pathogenesis of cutaneous fungal infection. For a better understanding of immune reactions in each series of cutaneous fungi, we also mentioned the importance of fungal cell components, which are recognized by immune cells and are responsible for the development of immune responses against cutaneous fungal infections.

### 1.1. Th17 Cells and Antimicrobial Action against Fungi

Inflammatory diseases such as rheumatoid arthritis, psoriasis, and inflammatory bowel disease are sometimes exacerbated by lymphocytes [14,15,16,17]. IL-17-producing cells are related to inflammation induced by the synergistic effect of certain inflammatory cytokines, including IL-23 and TNF-α, and antigen-presenting cells, following the upregulation of transcription factor RORγt [18]. In the development of IL-17-producing CD4^+^ cells, TGF-β and IL-6 are required for the differentiation from naïve T cells [19]. This developmental axis overlaps with Treg. While the origin of Th17 differentiation is intrinsically linked with Treg due to their common requirement of TGF-β signaling, Th17 differentiation also require IL-6 signaling [19].

IL-17-producing cells are recognized in Th17, Tc17, and γδT cells. Th17 cells are the main source of IL-17 in human skin and has been identified the pathogenetic role in various inflammatory skin diseases, such as atopic dermatitis and psoriasis [20,21]. In addition, CD8^+^ cells are abundantly increased in the lesional inflammatory skin, and these cells produce IL-17, namely Tc17 [22,23,24]. γδT cells are the main source of IL-17 in mice involved in the pathogenesis of inflammatory skin diseases, especially psoriasis [25,26,27,28,29].

**Figure 1:**
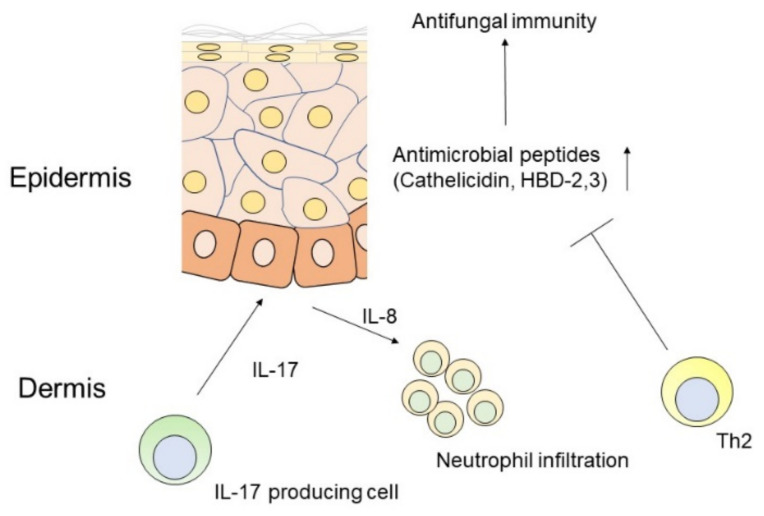
Scheme of antifungal immune responses mediated by IL-17 in the skin.

IL-17 plays a vital role in host defense against microorganisms through the production of antimicrobial peptides to promote microbial homeostasis and neutrophil recruitment [30] (Figure 1). Consistently, dysfunction of IL-17 signaling is closely associated with increased morbidity during microbial infection [31]. Predisposing host immune conditions, such as epithelial barrier disruption and cutaneous inflammation of the skin in atopic dermatitis, are closely related to the pathological function of commensal fungi-specific Th17 cells [32].

IL-17 drives IL-8 mediated neutrophil migration and antimicrobial peptide production, such as cathelicidin and human β-defencins (HBDs) by keratinocytes, which are impaired by Th2 cytokines.

### 1.2. Pattern Recognition for IL-17 Induction against Fungi

**Figure 2:**
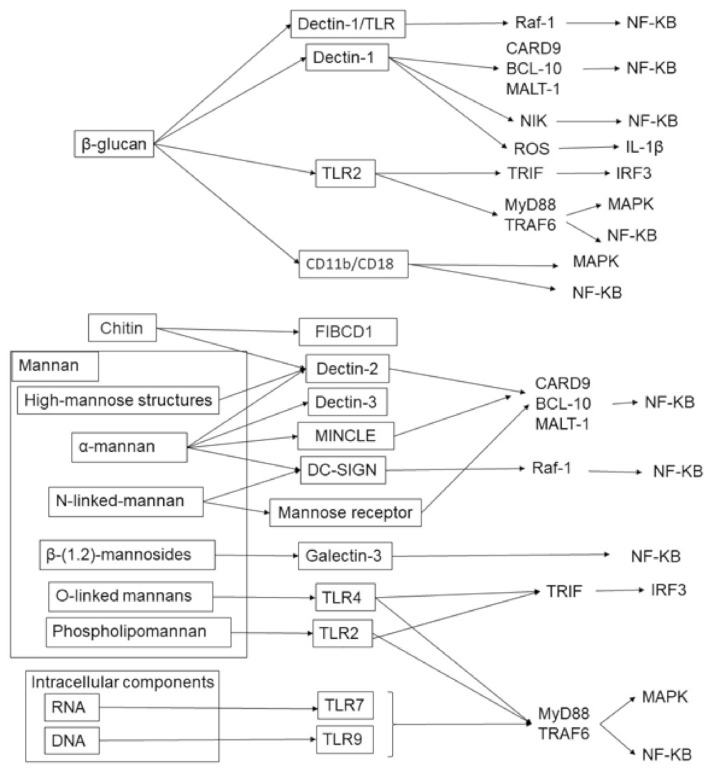
Fungal cell components and their receptors in humans.

The fungal cell wall is composed of three component matrices: Mannans, glucan, and chitin [33] (Figure 2). The outer layer consists of mannans such as O-linked and N-linked mannose polymers. In contrast, chitin is placed at the innermost layer of the cell wall and is connected with β-glucan to establish hydrogen bonds for constructing an inner layer network of microfibrils. Host innate immunity requires pattern recognition receptors (PRRs) to induce an immune response against fungi, namely pathogen-associated molecular patterns (PAMPs) (Figure 2). Four major PRRs have been identified: C-type lectin receptors (CLRs), Toll-like receptors (TLRs), retinoic acid-inducible gene I (RIGI)-like receptors (RLRs), and nucleotide-binding oligomerization domain (NOD)-like receptors (NLRs). TLR2, TLR4, and TLR9 can recognize phospholipomannan (PLM), and TLR7 is a receptor for single-stranded RNA, which is an intracellular component of fungi [34]. TLRs exhibit important signaling adaptors, such as MyD88 and TRIF. MyD88 combines almost all TLRs to drive downstream pathways, including NF-κB. Recent studies have revealed that the receptor for chitin, a cell wall component, contributes to the suppression of inflammatory responses. Further, inflammatory cytokine production is suppressed by FIBCD1-mediated mechanisms in lung epithelial cells [35]. Epithelial cell-specific FIBCD1-transgenic mice showed decreased fungal infection and impaired fungal-driven inflammatory responses in the intestine [36].

The cell components are mainly divided into cell walls and intracellular components, and there are various pattern recognition receptors and their downstream signaling pathways. The cell wall is composed of glucan, mannan, and chitin, and the intracellular components are RNA and DNA. These components drive specific pattern recognition receptor-mediated inflammatory immune responses.

PAMPs also enable the initiation of IL-17-producing cell development. For example, deficiency in the adaptor TRIF, which is downstream of TLR signaling, results in failure of IL-17-producing cell development in response to fungal infection. Dectin-1 is a receptor for β-glucan and can activate IL-17 production. In the skin, Langerhans cells recognize fungal pathogens, which drive TLR/MYD88 and dectin-1-dependent pathway and subsequently produces IL-6 mediated IL-17-producing cell development [12,37]. Blockages of IL-17 and IL-23 reduce chemokine and antimicrobial expression, and increase the risk of systemic fungal burden [38,39]. Therefore, it is assumed that humans have developed immune reactions against fungal cell components to eliminate infection and as the host immune response, and that IL-17 plays a vital role in the host defense against fungal infection.

### 1.3. IL-17 and Antimicrobial Action in the Skin

The main sources of antimicrobial peptides in human skin are keratinocytes, sebocytes, eccrine epithelial cells, mast cells, and neutrophils. Keratinocytes produce various antimicrobial peptides such as human β-defensins (hBDs), LL-37, psoriasin, RNase, lactoferrin, lysozyme, SLP, elafin, α-MSH, catestatin, and calprotectin. hBDs and LL-37 are also produced by eccrine epithelial cells, sebocytes, and mast cells. These antimicrobial peptides, especially defensins and LL-37 are produced in response to signal mediated by pattern recognition receptors, such as TLRs. Among antimicrobial peptides, hBD-2, hBD-3, LL-37, and psoriasin are activated by IL-17-mediated inflammatory responses. For instance, topical application of a vitamin D analog decreases hBD-2 and hBD-3, accompanied with reduced IL-17 in patients with psoriasis [40]. Further, IL-17A enhances vitamin-D-mediated LL-37 expression in keratinocytes in vitro [41]. Keratinocytes in atopic skin upregulate hBD-2 in response to IL-17 [42]. IL-17A enhances transcript expression of S100A7/psoriasin in human keratinocytes [43]. However, these antimicrobial peptides have limited expression under Th2-predominant skin conditions with co-expressed IL-4/IL-13, despite the presence of sufficient IL-17 production in the skin [42]. This inhibitory action against antimicrobial peptides explains the preferential colonization of the skin in atopic dermatitis with *S. aureus* and fungal infections, despite increased IL-17 production in the skin.

### 1.4. Skin Fungal Infection and Th17 Cells

Because the skin is the largest surface and an open field for the external environment in the human body, various microorganisms are transmitted from the external environment and from other humans and/or animals with easy access to the skin. To gain a better understanding of the role of IL-17-producing cells in host defense against cutaneous fungal infection, we have summarized the characteristics and pathogens of each series of cutaneous fungal infections and the actual impact of IL-17-producing cells.

## 2. Candidiasis

Candidiasis is a fungal infection by *Candida* species that is localized on the skin and mucosal surfaces; it is characterized by white patches on the tongue or other areas of the mouth and throat. Under immune-compromised host conditions, candidiasis becomes invasive and spreads to systemic organs. The cell wall of *Candida sp.* mainly consists of chitin, glucans, and mannans. Among them, mannans and mannoproteins are the most important for activating host defense. Other polysaccharides in *Candida albicans* (*C. albicans*), such as glucans and chitin, are much less immunogenic than mannans [44]. Mannans also induce immunosuppressive action by enhancing Treg function [45]. *C. albicans*-derived β-glucan exacerbates inflammatory cytokine production in macrophages by Dectin-1/TLR2 [46,47], SCARF1 [48], and CD36 [48]. In addition, mannan in *C. albicans* is recognized by TLR2 [49], TLR4 [47], mannose-binding lectin [50], galectin-3 [51], Dectin-2 [52], Dectin-3 [53], DC-SIGN [54], and mannose receptor [55]. The intracellular components of *C. albicans* such as RNA and DNA, are also recognized by TLR7 [34] and TLR9 [56], respectively.

In mouse skin, γδ T cells are the major source of IL-17A [26,27]. These cells are also induced under invasive *C. albicans* infection and are required for resistance against pathogens [57]. CD301b^+^ dermal dendritic cells are required for the IL-17A induction from dermal γδ T cells, and resistance to *C. albicans* requires IL-23 production. Sensory neurons are also involved in these mechanisms, and depletion impairs the elimination of *C. albicans* [57]. Candida-specific TCR transgenic mice reactive to *Candida*-derived endogenous antigens access the draining lymph nodes and are directly presented by migratory dendritic cells [58]. IL-17 is increased during the initial phase of cutaneous infection and is correlated with the rapid elimination of *C. albicans*. Inhibitory cytokines such as IL-10 and TGF-β are increased in the later phase and prevent exacerbation of inflammation in the skin [59]. In mice, γδ T cells are the main IL-17-producing cells in the initial phase after *C. albicans* infection; in the later phase, IL-17 production gradually shifts to the predominant αβ Th17 effector T cells. Afterward, the majority of *C. albicans*-reactive IL-17-producing T cells become tissue-resident memory CD4^+^ T cells. These CD4^+^ T cells acquire CD69 and CD103 expression for localization to the papillary dermis [60].

It has also been reported that IL-17 is essential for anti-fungal immunity mediated by the pattern recognition in human. An autosomal recessive form of susceptibility to chronic mucocutaneous candidiasis is associated with homozygous mutations in CARD9 [61,62]. Recurrent fungal infection is observed with CARD9 mutation, which is related to reduced Th17 cells. CARD9 deficiency results in an impaired immune response mediated by Dectin-1 [62]. Autosomal recessive deficiency of interleukin-17 receptor A (IL-17RA) and autosomal dominant deficiency of the cytokine interleukin-17F (IL-17F) impairs the responses to IL-17A and IL-17F. In contrast, IL-17F deficiency partially displays antifungal activity [63]. Autoimmune polyendocrinopathy-candidiasis-ectodermal dystrophy (APECED) with chronic mucocutaneous candidiasis shows neutralizing autoantibodies against Th17 cytokines and significant defects in IL-17F and IL-22 production [64]. Chronic mucocutaneous candidiasis with autosomal recessive IL-17RC deficiency leads to impaired cellular responses to IL-17A and IL-17F [65]. Signal transducer and activator of transcription 1 (STAT1)-gain-of-function mutations or heterozygous missense mutations in STAT1 result in severely impaired Th17 responses [66,67]. Impaired Th17 is also observed in patients with loss-of-function STAT3 mutations (autosomal dominant hyper-IgE syndrome; AD-HIES) with recurrent oral fungal infections [68].

## 3. Dermatophytosis

Dermatophytosis is a fungal infection of the skin that is typically caused by *Trichophyton*, *Microsporum*, and *Epidermophyton* species. It often causes ring-form scaly erythematous skin, particularly with *Microsporum* species. Healthy subjects are also at the risk of infection due to contact sports and obesity. The cell wall of *Trichophyton* is enriched in mannan, which can activate immune reactions by itself [49]; in contrast, mannan from the cell wall of *Trichophyton rubrum* has the potential to inhibit cell-mediated immunity and keratinocyte proliferation [69,70].

Superficial dermatophytosis presents in the stratum corneum and cannot directly make contact with immune cells in the dermis. Therefore, some possible anti-fungal immune responses have been postulated [69]. In humans, an increased number of Th17 cells is observed in the peripheral blood and skin of patients with dermatophytosis [71,72]. Consistently, innate immune disrupted diseases, such as adult-T cell leukemia/lymphoma (ATLL), commonly exhibit superficial dermatophytosis among cutaneous mycotic infections [73]. The frequency of Th17 cells in peripheral blood is reduced in patients with ATLL [74]. Further, the epidermal expression of hBD-2, LL-37, and S100A7/psoriasin is decreased in patients with ATLL and dermatophytosis [74,75], indicating that the secretion of Th17-mediated antimicrobial peptides by keratinocytes is reduced, leading to the frequent occurrences of dermatophytosis.

In an animal model of superficial skin infection with *Microsporum canis*, mild inflammation in the skin was observed with a Th17-mediated immune response by Langerin^+^ cells [76]. IL-17 deficiency allows *M. canis* to colonize the epidermis and exacerbate skin inflammation through an IFN-γ-mediated response. Dectin-1 and Dectin-2 deficiency result in inadequate production of inflammatory cytokines in response to *T. rubrum* infection and impair its elimination [77]. In addition, mice lacking IL-1R show decreased IL-17 production in response to *T. rubrum* [78]. STAT1 gain-of-function mutation also facilitates severe dermatophyte infection in the skin with impaired Th17 responses in the peripheral blood [66].

## 4. Malassezia

Malassezia is localized on the skin surface of many animals including humans. Representative clinical skin diseases include pityriasis versicolor and Malassezia folliculitis. In pityriasis versicolor, *Malassezia* species, especially *Malassezia globosa*, can cause hypopigmentation or hyperpigmentation on the trunk and other regions of the human body. Malassezia folliculitis is often observed in adolescents and is characterized by follicular erythematous papules localized on the chest, back, and upper arms. *Malassezia* species have β-glucan-enriched cell walls [79], and drives responses mediated by TLR2 [80], Dectin-1 [81], Dectin-2 [81], MINCLE [82], mannose receptor [83], and TLR9 [84].

T cells in the peripheral blood and skin of patients with atopic dermatitis sensitized to *Malassezia* secrete IL-17 and IL-22 [85], possibly due to autoimmunity and cross-reactivity between a fungal antigen and human thioredoxin. *Malassezia* metabolites also enhance the expression of IL-36γ in human epidermal keratinocytes in vitro, which induces IL-17 production [86].

In an experiment with mice, IL-17A and IL-22 induction is observed in response to *M. pachydermatis*-colonized skin, which prevented fungal growth on the skin [87]. Further, inhibition of the IL-23/IL-17 axis was found to impair *Malassezia*-specific immunity in the skin and to disrupt the fungal clearance mediated by CARD9 or MYD88.

## 5. Sporotrichosis

Sporotrichosis is a disease caused by *Sporothrix schenkii* (*S. schenckii*) and usually affects the skin. Because this species exists naturally in soil and plants, it usually affects agricultural workers and enters through small skin wounds to cause infection [88,89]. Sporotrichosis growth is relatively slow, and a long time is required for the first symptoms to appear, about 1 to 12 weeks after the initial exposure to *S. schenckii* [90]. Immune-compromised hosts sometimes develop serious complications that can spread to systemic organs. The cell wall of *S. schenkii* is recognized by Dectin-1 [91] and TLR4 [92] in macrophages and by TLR2/4 in keratinocytes [93].

*S. schenckii* activates different immune profiles during host defense. Upon subcutaneous injection, *S. schenckii* enhances IL-17 production [94]; however, this peak gradually decreases in the later phase [95]. Dectin-1 and IL-17 are not essential for *S. schenckii* clearance, suggesting that different molecular recognition pattern of *S. schenckii* might be targeted to exert antifungal action in cutaneous immunity [95].

Systemic dissemination of *S. schenckii* by intraperitoneal administration results in an enhancement of Th17 response [96]. Neutralizing IL-23 antibody treatment, which blocks downstream IL-17 production, is closely associated with an impaired capacity to control *S. schenckii* infection [96]. While fungal loads are increased by anti-IL-23 antibody treatment, this does not affect survival against infection. Th17 is essential to eliminate *S. schenckii* infection in the initial phase, but not for survival against *S. schenckii* infection.

The NLRP3 inflammasome is related to the innate immune recognition of *S. schenckii* to exert an adaptive immune response for protection against infection [97]. The NLRP3 inflammasome appears to be critical for IL-17 release. NLRP3 deficiency results in susceptibility to infection, suggesting that NLRP3-mediated responses contribute to the development of host protection against *S. schenckii* infection. Furthermore, *S. schenckii*-induced IL-17 production is a TLR2 independent mechanism, and the absence of a Th1 response in TLR2 deficiency is concomitant with IL-17 production [98].

## 6. Chromoblastomycosis

Chromoblastomycosis is a chronic fungal infection in the subcutaneous tissue and is most commonly observed in tropical climates; it is caused by *Fonsecaea monophora*, *Fonsecaea pedrosoi* (*F. pedrosoi*), *Phialophora verrucosa*, and *Cladophialophora carrionii*. Chromoblastomycosis is caused by various fungal infections in subcutaneous lesions, and its clinical prognosis is not favorable without treatment. In contrast, it is intractable and persistent with the current treatments. Chromoblastomycosis pathogens activate TLR2/4 [99], NLRP3 [100], Dectin-1 [101], Dectin-2 [102], and MINCLE [101]. The pathogen component chitin suppresses the Th1 immune response [103].

Chromoblastomycosis in human skin shows high expression of IL-17 [104]. In an experimental chromoblastomycosis infection animal model, *F. pedrosoi* hyphae-injected skin shows that T cells are skewed into Th17-and Th1 dominant phenotypes [102]. Th17 cells are increased in the injected skin lesion during the early phase, followed by a Th1 dominant condition in the later phase. IL-17-neutralizing treatment decreased fungal elimination. In contrast, the cell wall of a chitin-like pathogen component in chromoblastomycosis suppresses IL-17 via Dectin-1 [105]. In addition, a recent study identified that the non-recognition of *F. pedrosoi* by TLR might also play a role in the defective production of proinflammatory cytokines [106]. This might be involved in the pathogenesis of chronic infections in chromoblastomycosis.

## 7. Mycetoma

Mycetoma is a chronic subcutaneous infection by eumycetoma fungi, especially *Madurella mycetomatis* and *Medicopsis romeroi*, which infiltrate the body through the skin surface, and is commonly observed in the foot; it is characterized by a subcutaneous solid mass and eventually invades the underlying bone. *Pseudallescheria boydii* is also a mycetoma pathogen and is recognized by TLR2 [107] and TLR4 [108], to produce proinflammatory cytokines in macrophages.

However, these findings do not reflect the actual impact of IL-17 resistance against mycetoma. Immune responses such as antimicrobial peptides may not be effective against mycetoma. Consistently, a previous study showed that hBD2 expression could not be detected in the skin of patients with mycetoma [109], suggesting that some innate immunity dysfunction might be the reason underlying chronic mycetoma infection, despite sufficient IL-17 production in the skin.

## 8. Paracoccidioidomycosis

Paracoccidioidomycosis is an acute to chronic fungal infection caused by *Paracoccidioides* species, especially *Paracoccidioides brasiliensis* (*P. brasiliensis*) and *Paracoccidioides lutzii*. It is commonly observed in areas of Central and South America. Paracoccidioidomycosis is characterized by ulcers and granulomatous lesions on the skin, which generally occur several weeks or months to years after the initial exposure to the fungus. Pathogens of paracoccidioidomycosis are recognized by TLR9 [110], TLR4 [111], TLR2, 4, Dectin-1 [112], DC-sign [113], and Galectin-3. Galectin-3-deficient macrophages exhibit higher TLR2 transcript levels and IL-10 production than wild-type macrophages after stimulation with *P. brasiliensis* antigens [114].

In vitro analysis indicated that *P. brasiliensis*-infected antigen presentation cells undergo Th17 differentiation mediated by Dectin-1 [115], TLR4 [115], NLRP3 [116], and IFN-β/Caspase11/IL-1α [117]. Antigen presenting cells also require IDO1 for Th17 differentiation [118]. Further, IDO1-deficient mice show decreased their survival after intratracheal injection of *P. brasiliensis.*

Few studies have focused on the cutaneous infection of *Paracoccidioides*. Paracoccidioidomycosis shows epidermal hyperplasia and well-organized granulomas with a high frequency of IL-17-expressing cells [119]. Intratracheal injection of *P. brasiliensis* infection promotes the IL-17 production in the lung in a Myd88-dependent manner [120]. *Paracoccidioides* infection in the lung induces IL-17 production mediated by Dectin-1 [121], Caspase 11, IL-1α [117], and NALP3 (NLRP3) [122], which are required for fungal clearance. Thus, IL-17 is responsible for anti-fungal action against *Paracoccidioides* infection; however, the related cutaneous immune responses are largely unknown.

## 9. Coccidioidomycosis

Coccidioidomycosis is a fungal skin disease caused by *Coccidioides immitis* or *Coccidioides posadasii* (*C. posadasii*) and is observed in certain parts of the United States, such as California, Arizona, Texas, and northern Mexico. Coccidioidomycosis is characterized by skin ulcers, abscesses, and bone lesions, and sometimes involves systemic organ dysfunction, such as inflammation of the heart and brain, which can lead to fatality. TLR2, TLR4, and Dectin-1 are responsible for eliminating *Coccidioides* mycosis [123]. Thus, vaccination against *C. posadasii* infection depends on innate sensing by Dectin-1 and Dectin-2 [124].

In the skin, IL-17 contributes to the suppression of the *Coccidioides* growth under certain conditions. Although IFN-γ receptor-deficient mice show the burden of *Coccidioides* infection, IL-17A-depleted IFN-γ receptor mice show significantly increased growth of *C. posadasii*. This indicates that IL-17 mediated immune responses are required protection against subcutaneous *C. posadasii* infection under IFN-γ-insufficient conditions. Therefore, IL-17 might play a more important role in immunity against *C. posadasii* under Th2-dominant conditions in the skin [125].

In contrast, the IL-17 mediated antimicrobial action differs in other organs. For instance, IL-17A-deficient mice exhibit partial clearance of *Coccidioides* from the lungs after intranasal injection [126]. IL-17 receptor A-deficient mice show sustained *Coccidioides* infection after immunization, which decreased their post-challenge survival [127]. 

## 10. Conclusions

This review summarizes the updated role of Th17 cells in antimicrobial action against cutaneous fungal infections. Almost all cutaneous fungal infections exhibit enhanced Th17 reaction in the initial phase; however, this reaction is impaired in the later phase. Although the underlying reason remains unclear, this explains the plasticity of Th17 cells under the influence of IL-23, leading to inhibition of IL-17 production and induction of IFN-γ and/or GM-CSF production [30]. The detailed mechanism needs to be clarified, because this mechanism might contribute to intractable chronic fungal infection. Research on *Candida* infection is well-established and updated in the field of anti-fungal dermatology. Other fungal species also need to be studied in the same manner, because IL-17 responses for the elimination of fungi in skin infection are different for several species of fungi.

## Data Availability

Not applicable.
